# Improvement in the Carbonation Resistance of Construction Mortar with Cane Bagasse Fiber Added

**DOI:** 10.3390/ma14082066

**Published:** 2021-04-20

**Authors:** William A. Talavera-Pech, Diana Montiel-Rodríguez, Josefa de los A. Paat-Estrella, Ruth López-Alcántara, José T. Pérez-Quiroz, Tezozomoc Pérez-López

**Affiliations:** 1 Centro de Investigación en Corrosión, Universidad Autónoma de Campeche, Av. Héroe de Nacozari No. 480, San Francisco de Campeche, Campeche 24079, Mexico; watalave@uacam.mx; 2 Facultad de Ciencias Químico Biológicas, Universidad Autónoma de Campeche San Francisco de Campeche, Campeche 24085, Mexico; hi_dian@hotmail.com (D.M.-R.); josapaae@uacam.mx (J.d.l.A.P.-E.); 3 Facultad de Ingeniería Civil, Universidad Veracruzana, Circuito Gonzalo Aguirre Beltrán s/n Zona Universitaria, Xalapa C.P. 91090, Mexico; 4 Centro de Investigaciones Biomédicas, Av. Agustín Melgar s/n. Universidad Autónoma de Campeche, San Francisco de Campeche, Campeche 24039, Mexico; rutlopez@uacam.mx; 5 Instituto Mexicano del Transporte, Km 12+000, Carretera Estatal No. 431 “El Colorado-Galindo”/Parque Tecnológico San Fandila/Mpio, Pedro Escobedo, Querétaro 76703, Mexico; jtperez@imt.mx

**Keywords:** sugarcane bagasse fiber, carbonation, mortar, FTIR analysis

## Abstract

In this work, sugarcane bagasse fiber, a waste product of agroindustry, was added to mortar mixes at different proportions looking to seal porosities so as to improve the resistance of concrete to carbonation and to improve its mechanical properties. To evaluate the behavior of bagasse fibers in the alkaline media typical of mortars, bagasse fibers were subjected to solutions with alkaline pH values, and their chemical structure and morphological behavior was evaluated using FTIR (Fourier transform infrared spectroscopy) and SEM (Scanning Electron Microscopy). Using mortar cylinders in an accelerated carbonation chamber to obtain results in short lapses, the compressive strength and the carbonation were evaluated. The FTIR analysis results indicate that pH values of 11 and 12 causes a delignification, while at pH 9 and 10, a swelling of the molecule occurs because of the addition of hydroxyl ions, behavior that is confirmed with SEM images. A clear effect of the fiber addition on the performance of concrete was observed as the carbonation front of 35 mm for the sample without fibers was reduced to 2 mm for the sample with 2% fiber addition, resulting in an increase of 5 MPa in compressive strength. These results indicate that in the range of mortar pH, chemical changes occured over the sugarcane surface that could cause the growth of fibers and could partially seal the porosity in the mortars, thus enhancing its performance.

## 1. Introduction

Concrete is the most widely used construction material today, because of its chemical and mechanical properties that make it more resistant to the environment and provide great durability. However, it is not an inert material, as the pore solution formed inside it can react with the carbon dioxide (CO_2_) of the atmosphere, which generates CaCO_3_ and results in a process known as carbonation [1]. As a consequence of carbonation, the pH of the pore solution decreases, generating conditions for a corrosion process to start on the embedded steel reinforcement. For this reason, a criterion of durability is to design concrete with a minimum porosity in order to slow the advance of carbonation.

Various ways to improve the durability properties of concrete have been tried. One of them is the incorporation of waste materials, such as glass [2], PET containers [3], tile and sanitary ceramics [4], clay bricks [5], tires and rubber [6], concrete waste [7], farming waste residues [8,9], and metal slag [10], which provide a beneficial environmental effect by reusing materials that have already completed their utility cycle. The results of the above-mentioned studies show the possibility of using these waste materials as alternatives in construction, as these materials enhance the performance of concrete in terms of its compressive strength, durability, water absorption, and elasticity, among others [11]; In addition, their use reduces the extraction and use of conventional materials, which not only minimizes cost, but also provides security in terms of their properties and resistance needs according to their uses [12,13]. However, some do not present significant improvements and there are concerns about their workability, ASR (Alkali Silica Reaction) expansion, and tensile strength decrease [14].

Reinforced concrete is currently the most widely used construction material. However, it is a great generator of CO_2_ emissions to the atmosphere during the cement manufacturing process. This contributes to global warming, as it is a greenhouse gas that contributes to the current climate impact [1,15,16,17]. Because agriculture generates around 20–30% waste, it has been proven to be a material that can be reused for ecological purposes. Recently, agricultural waste materials have been used as substitutes for reinforced concrete components. For example, flax fibers have shown that they can improve mortar workability [18], while coconut shell can produce light concrete with an enhanced compressive strength [19]. In the state of Campeche, 10,500 hectares (ha) are cultivated and 427 thousand tons (t) of cane are produced, which, when ground, generate 46,000 tons of sugar. Its cultivation ranks third in the planted area after corn and rice and provides sustenance for 13,500 families [20]. However, one of the waste products obtained at the end of the sugarcane juice production process is bagasse fiber, which is incinerated in mill boilers as a fuel, causing damage to the environment as a result of the gas emissions that are released into the atmosphere.

Several authors have attempted to incorporate sugarcane bagasse in the form of fibers in order to improve the properties of reinforced concrete mixtures [21,22,23]. They have observed some advantages for the incorporation of these fibers, such as an increase in toughness and impact resistance. In a fresh state, it allows for controlling the plastic shrinkage in the setting period. Sugarcane bagasse is composed of carbohydrate-type biopolymers with approximate compositions of 50% cellulose, 25% hemicellulose, and 25% lignin [9]; such biopolymers, in turn, are composed of polysaccharides. In its structure, lignin is embedded in the structures of cellulose and hemicellulose, acting as a “glue” between them, through the formation of ester and ether-type bonds with those structures [24]. In the aforementioned tests of bagasse additions to concrete, one of the results is an increase in the compressive strength [25,26]. Still, there are no recorded experimental data about how this is related to the degradation of sugar cane bagasse fibers at alkaline pH values such as those that occur in concrete in its curing stage. Omoniyi et al. modified fiber properties using alkaline treatments in order to find better conditions of durability [27]. However, they did not test a variation of pH from 12 to 8, which might be preset during the concrete carbonation process. In this work, surficial changes over fiber such as pH variation, show that at values of 12 and 11, the fiber is affected. In the pH range of 10 to 8, sugar cane fiber does not suffer from chemical or structure changes. In addition to the long-term performance, the reactions and structural changes that occurr at pH 12 and 11 contribute to reducing the carbonation advanced into concrete.

To the best of our knowledge how the alkaline media of mortars can chemically and morphologically affect the cane bagasse fibers and the relationship that this has with the improved properties of concrete with the addition of fibers have not been evaluated. Because of the above, this project aims to determine the physical (structural) and chemical changes in sugar cane bagasse fibers subjected to pH values typical of mortars, and to explore how these changes can affect the properties, in terms of compressive strength and carbonation resistance, of mortar samples made with the addition of cane bagasse fibers at different percentages, giving added value to this waste. This is an initial study on the addition of sugar cane fiber in order to reduce the rate of carbonation. Although the results are for a short time (90 days), is a quality test to assist with recommendations for medium- and long-term use.

## 2. Materials and Methods

### 2.1. Sugarcane Bagasse

#### Preparation of Sugarcane Bagasse Fibers

A bagasse sample was taken at La Joya sugar mill located in Sihochac, Municipality of Champotón, Campeche, Mexico. Three sacks of fiber were mixed to obtain a representative distribution of the material. Subsequently, by applying the method of quartering, a 2 kg sample was obtained to prepare the test specimens, which were subjected to two washes with water from the municipal intake, and one wash with purified water to remove possible traces of sugar.

### 2.2. Degradation Studies of Sugarcane Bagasse

#### 2.2.1. Infrared Spectroscopy Analysis (FTIR)

Dry samples of approximately 1 g of sugarcane bagasse were weighed and exposed to solutions with pH values of 12, 11, 10, 9, and 7 as controls. Solutions were prepared with reagent grade NaOH and distilled water. After periods of 15 and 30 days of exposure, the samples were separated from the solutions by filtration.

To know the chemical composition of the materials before and after the exposure periods, Fourier transform infrared spectroscopy (FTIR) tests were carried out using the attenuated total reflectance (ATR) technique in a spectral interval of 4000 to 400 cm^−1^, with an average of 100 scans at a resolution of 4 cm^−1^.

The above was done to get the approximate effect of the pH variation on the chemical stability of bagasse, with the knowledge that freshly cast concrete reaches a pH of 12 to 12.5 and, when carbonated, the pH can reach values close to 9.

#### 2.2.2. Analysis by Scanning Electron Microscopy (SEM)

Scanning electron microscopy images were obtained for the samples subjected to the degradation described in the previous section. The materials were chosen in stages and at strategic pH values in order to monitor their degradation. A Hitachi Flexsem 1000 scanning electron microscope (Hitachi, Tokyo, Japan) at 20 kV and 60 Pa was used for this purpose.

### 2.3. Performance Tests of Mortar Samples

Conventional cylindrical mortar samples with a 15 cm diameter and 30 cm height were prepared for the compressive strength tests. A 1:2:3 ratio of water/cement/fine aggregate was used, and the water/cement ratio was 0.5. A quantity of 35 mL of fluidizing additive (lignosulfonate), namely, DISPERCON AL-100 trademark PROCONSA (Cuautitlán, Mexico), was used for each liter of water (0.0175 water/cement). Dry sugarcane bagasse was added to the mortar mixture in proportions of 0.5, 1.0, and 2.0% weight of cement.

Composite Portland cement (CPC) according to NMX-C-414-ONNCCE-2017 [28], Maya commercial brand (Mérida, México); purified water; and local fine aggregate were used. All of the components of mortar are reported in Table 1. The fine aggregate used was obtained from local crushed limestone, characteristic of that found in the Yucatan peninsula, with a maximum size of 3.35 mm. Sugar cane fibers of 2.5 to 3 cm in length were added according to % weight of cement. The mixture process consisted of mixing the fibers with the fine aggregate; afterward, they were combined with cement, taking care to obtain a homogeneous dispersion; each step was done in less than 5 min. Finally, 50% of the water with the fluidizing additive dissolved was added for the initial mix. The rest of the water with the water reducer was added in order to complete the required quantity. Each of the steps were carried out with a shovel until a homogeneous mix was obtained.

#### 2.3.1. Compressive Strength

**Figure 1:**
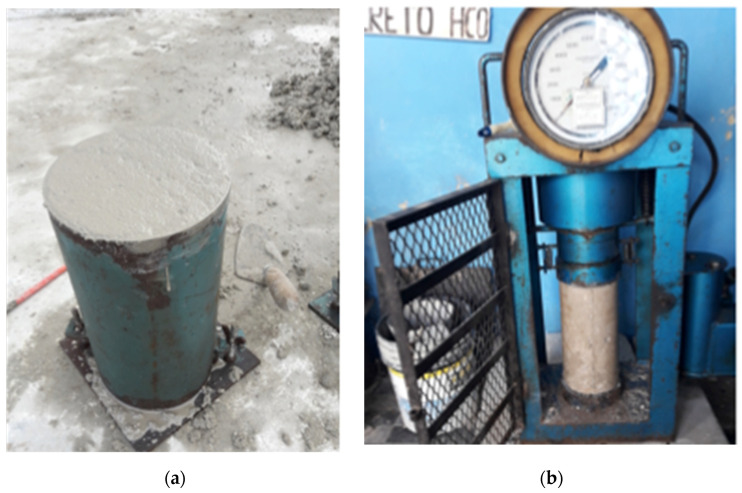
(**a**) Cylinder sample for compressive resistance and (**b**) compressive resistance assay.

Cylinders with a 15 cm diameter and 30 cm height were cured by immersion in a saturated Ca(OH)_2_ solution, according to the NMX-C−159-ONNCCE-2016 [30] standard. The compressive strength was measured after 7, 14, 28, and 90 days of curing, as indicated in the NMX-C-083-ONNCCE 2014 standard [31]. Sample elaboration and compressive resistance tests were carried out as shown in Figure 1a,b.

#### 2.3.2. Carbonation

For the carbonation tests, cylindrical samples with a 7.5 cm diameter and 15 cm height were made, with the same bagasse addition rates as those used for the compression resistance (0, 0.5, 1, and 2%). They were placed in an accelerated carbonation chamber with the function of maintaining the experimental conditions at 25 ± 2 °C temperature; 65 ± 5% relative humidity, and 3% CO_2_ concentration. The carbonation front was measured at 90 days of exposure, as indicated in the NMX-C-515-ONNCCE-2016 standard, by using a phenolphthalein acid/base indicator [32].

#### 2.3.3. Volume of Permeable Voids

The standard ASTM C 642 2006 [33] procedure to determine the volume of permeable voids in the mortar samples was used. To measure the weight of sample, a balance Precisa XB 2200C (Precisa, Dietikon, Switzerland) model with a range of 2200 g and 0.01 g as the sensibility was used. For the drying specimens, a laboratory drying oven (RIOSSA Monterrey, México) with a 10 to 250 °C range was utilized. For the wet steps, distilled water was used.

## 3. Results and Discussion

### 3.1. Degradation Studies of Sugarcane Bagasse

#### 3.1.1. Fiber Analysis by FTIR

**Figure 2:**
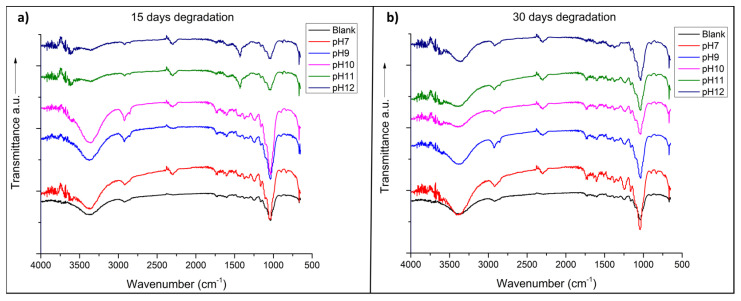
FTIR (Fourier transform infrared spectroscopy) spectra of the samples from the bagasse (blank) and subjected to different pH values: (**a**) 15 days of degradation and (**b**) 30 days of degradation.

In Figure 2a,b, the spectrum of each sample after 15 and 30 days of degradation is observed, respectively. The spectrum of untreated (blank) cane bagasse shows all the characteristic peaks of the cellulose, lignin, and hemicellulose, which are the main components of the bagasse fibers [34,35]. The peak at 3440 cm^−1^ corresponds to the stretching vibrations of the OH bonds; at 1730 cm^−1^, the peak corresponding to the stretching vibration of the C=O bond is found because of the ester and acetyl groups from either hemicellulose or to the ester type linkages of the carboxylic groups of the ferulic and p-coumaric acids contained in the lignin and/or hemicelluloses. The absorption peaks close to 1602 cm^−1^ and 1505 cm^−1^ correspond to the symmetric stretching vibration in the plane of the C=C bonds of the aromatic rings found in the lignin structures. There is a small peak between 1430 cm^−1^ and 1420 cm^−1^ is associated with the scissoring of the CH_2_ bonds present in cellulose. At 1375 cm^−1^ and 1320 cm^−1^ the absorptions of CH bending and OH bending in-plane are found. At a wavenlength of 1245 cm^−1^, the out-of-plane stretching vibration of the CO bond of the aryl groups present in the lignin is also found; while the peak at 1054 cm^−1^ corresponds to the asymmetric stretching vibration of the C-O-C bond, both characteristic of the glycosidic structures present in all of the components of sugarcane bagasse. Finally, at 902 cm^−1^, it presents a peak that is associated with the absorption of the β-glycosidic bonds between glucose units in cellulose [36,37,38,39,40].

In the spectra of degraded fibers at different pH values after 15 days (Figure 1a), the spectrum of the sample subjected to a pH value of 7 was very similar to the untreated sample, while a pH 9 and 10 were also similar to the previous two. However, there was an increase in the absorption of the band at 3440 cm^−1^, which indicates that, at these pH values, the OH groups were inserted into the molecular structures, specifically into the structures of the hemicellulose and lignin [41].

In the spectra of samples subjected to pH values of 11 and 12, there were clearly observable changes with respect to all of the previous samples, as the peaks at 1730 cm^−1^, 1602 cm^−1^, 1505 cm^−1^, and 1245 cm^−1^, were lost. The first was characteristic of the ester-type bonds that bind hemicellulose and lignin with cellulose fibers [34], while all of the others as mentioned above were characteristic of the molecular structures of hemicellulose and lignin. Additionally, these spectra maintain the characteristic peaks exclusively of cellulose, such as the peak at 902 cm^−1^ and a higher absorption for the peak at 1430 cm^−1^, which was also specific to cellulose.

Degradation at 30 days produced results very similar to those at 15 days. The spectra of samples subjected to pH 7, 9, and 10 remained practically unchanged, while the spectra of samples subjected to pH 11 and 12 lost their peak at 1430 cm^−1^, which is characteristic of cellulose; however, the peak at 902 cm^−1^ remained unchanged.

These results prove that at high pH values (11 and 12), the samples lose the characteristic lignin peaks, which correspond to delignification, while at pH values of 9 and 10, hydroxyl ions are added to the structure of the components of the bagasse fiber, which causes swelling of the molecules, hydrolysis of the ester bonds, and the breaking of the inter-molecular hydrogen bonds between hemicellulose and cellulose, causing hemicelluloses to be solubilized in water, separating from the cellulose fibers [42]. These results confirm the findings of Silva and Rodríguez, who mention that natural fibers immersed in Portland cement suffer degradation as a result of a high alkaline environment, which dissolves the lignin and hemicellulose chains, weakening the fiber structure [43]. Ramakrishna and Sundararajan observed that the same degradation of fibers immersed in Ca(OH)_2_ can be related to the crystallization of lime in the fiber pores [44].

#### 3.1.2. Analysis by Scanning Electron Microscopy (SEM)

**Figure 3:**
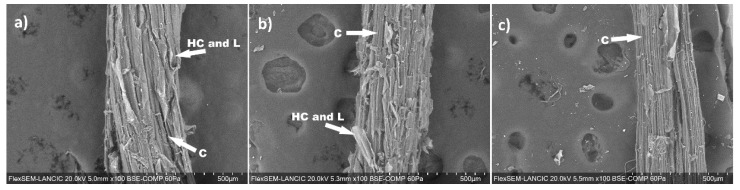
Electron microscopies of the samples of bagasse: (**a**) as obtained (blank), (**b**) at pH 7 after 15 days of degradation, and at (**c**) pH 12 after 30 days of degradation.

The SEM images of the blank and degredation samples were taken at representative pH values and times in order to observe the degradation of samples under such conditions. It is known that cane bagasse fibers are mainly composed of fibers of crystalline polymer cellulose (C on images). The other main components are hemicellulose (HC on images), which is an amorphous polymer, and lignin (L on images), which functions as a cementitious matrix between cellulose and hemicellulose [45]. All of these components can be seen in Figure 3a, corresponding to the untreated bagasse, in which the cellulose fibers composed of microfibrils are shown. Each of them is aligned in the direction of the axis. However, it is difficult to observe the fibers in detail, as they contain surface layers of non-fibrous components, corresponding to materials such as hemicellulose and lignin [40].

Figure 3b shows the micrograph corresponding to the sample subjected to pH 7 for 15 days, in which the same components are presented as in the untreated bagasse, although in this sample, the non-fibrous materials can be seen slightly more disordered and swollen because of the contact with the aqueous solution where they can gain hydroxyl ions. The micrograph presented in Figure 3c corresponds to the material subjected to pH 12 for 30 days. In this image, the cellulose microfibrils that make up the fiber can be more easily seen, because the lignin and cellulose were hydrolyzed and solubilized after these conditions of exposure, as corroborated by FTIR, which leads to defibrillation of the fibrils. Therefore, as there is no non-fibrous material covering the microfibrils, they are more exposed to deterioration [45].

### 3.2. Performance Tests of Mortar Samples

#### 3.2.1. Compressive Strength

**Figure 4:**
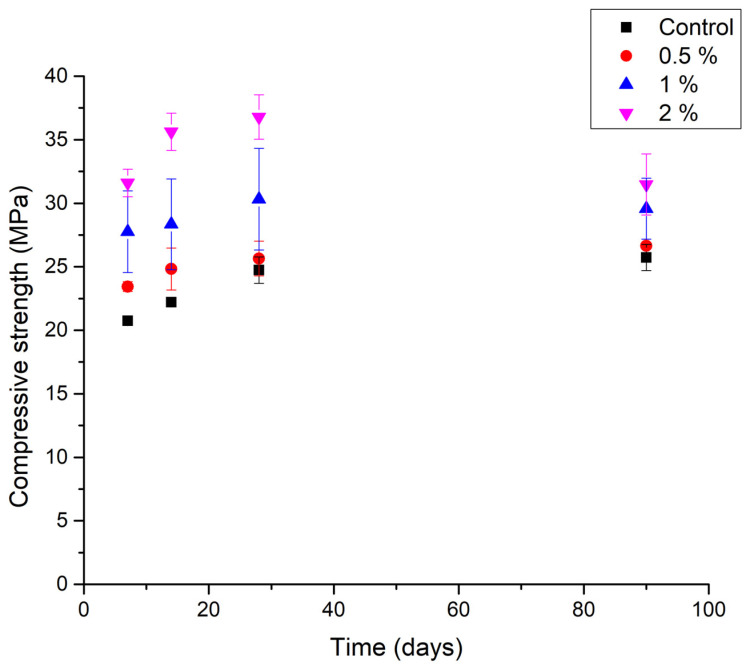
Compressive strength as a function of time and fiber proportion.

Figure 4 presents the results of the compressive strength monitoring at 7, 14, 28, and 90 days for this work. It is clearly seen that the addition of cane bagasse increases the compressive strength of the samples compared with the blank specimens. Maximum increments for the bagasse concentration were 12.5% for 0.5%, 33.84% for 1%, and 60.38% for 2%. This is because the fibers get trapped in the cementitious matrix and provide it with greater resistance. Brandt proposed that the fibers apport control above the opening and propagation of the microcracks as they are densely dispersed in a cement matrix [46]. Kaushik and Biswas found that at low levels of lignin, modified lignin can yield a high-performance concrete strength and grinding, and reduces the damage of external walls due to moisture and acid rain; additionally, they found that some selective lignin can improve the compressive strength of cement pastes [47].

The FTIR results show the depolymerization of lignin, which were modified to form derivatives with the characteristics mentioned above. Some authors of investigations into sugarcane bagasse treatments in alkaline media have found variations of components after reactions. Wunna et al. analyzed the decomposition of sugarcane bagasse in an alkaline pH, and found decomposition of lignin and hemicellulose [48]. Maryana et al. found that the crystallinity of cellulose decreased after alkaline sugarcane pretreatment, which is in line with decreasing their lignin level [49]. Chin et al. reported that the increasing trend of the total reducing sugar might be due to the lignin removal with the increased Ca(OH)_2_ (alkaline) concentration from 1% *v*/*w* to 2 *v*/*w*%, and the mechanism is said to be saponification of intermolecular ester bonds cross-linked with hemicelluloses and lignin [50]. 

In this work, at 2% of the bagasse, resistance after 90 days decreased, possibly due to the decomposition of fibers when exposed to an alkaline environment. However, this sample still had a better compressive strength, exceeding the control sample by 5 MPa at 90 days.

The densities of the composite mortar increased with increasing the fiber length and volume. The compressive strengths of the composite mortar increased at 2% fiber volume with lengths of 10 and 20 mm [51]. This effect sealed the porosities of mortar and decreased the advancement of the carbonation front.

At concentrations of 0.5, 1, and 2%, the tendency was to maintain a higher value than the control. Huerta and Martínez [52] mention that a mixture with an additional range lower than 2% improves resistance, and when using values greater than this percentage, there is a decrease in the compressive strength of the concrete. Osorio et al. [53] observed that the cane bagasse fiber used in the elaboration of compression-reinforced concrete imparts important mechanical properties to the compound, mainly to specimens with fiber additions between 0.5 and 2.5% regarding the total weight of the coarse aggregate. They propose that, at low amounts, the fibers remain attached to the cementitious matrix and increase its resistance.

Wegdan [13] reports that the highest compression strength was obtained with 0.5% sugarcane bagasse fiber. In the present work, the increase in the amount of fiber in the range from 0 to 2% corresponds to the increase in compression resistance.

#### 3.2.2. Carbonation

**Figure 5:**
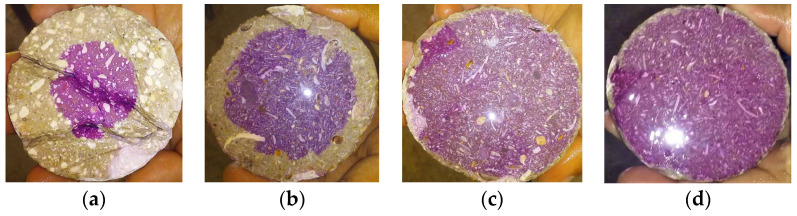
Carbonation of concrete samples: (**a**) control, (**b**) 0.05%, (**c**) 1%, and (**d**) 2%.

**Figure 6:**
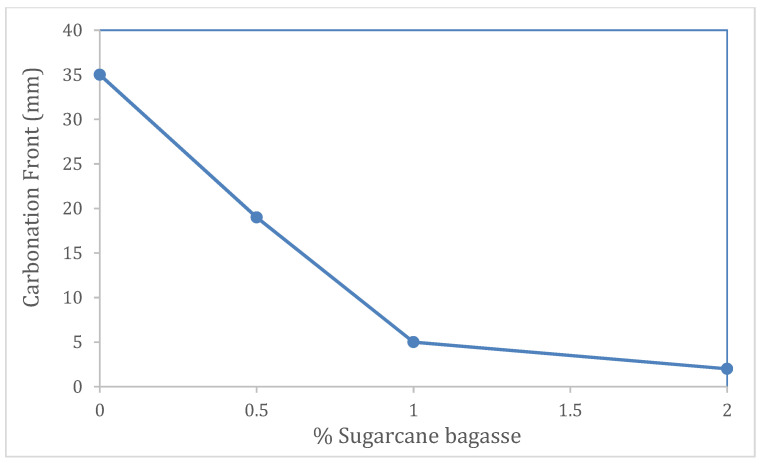
Progress of carbonation for the concrete samples with different amount of fibers added: control (0%), 0.5%, 1% and 2%.

Figure 5 exposes the results of the carbonation front at 90 days. The difference in the advancement of the carbonation front with the addition of fiber is significant. The decrease in the rate of the carbonation process is seen with the increase in the amount of sugarcane bagasse in the concrete mix. In Figure 6, the advance of carbonation is quantified, and the proportion is inverse to the amount of fiber added.

Figure 3a–c shows that the fiber presents greater degradation in an alkaline medium similar to the pore solution of concrete, as can be observed in the scanning electron microscopy section, which generated an expansion in the fiber components. In this regard, the authors propose that the unfolding of fiber components occurs in smaller parts, which have a hygroscopic capacity that favors an increase in volume, thus reducing the dimensions of concrete pores, generating their sealing, and therefore decreasing the advance of the carbonation.

Monteagudo proposed that when the pH decreases in a cement paste, the sequence of reactions that follow comprise the dissolution of the portlandite Ca(OH)_2_ and the precipitation of ettringite (3CaO-Al_2_O_3_-3CaSO_4_-31H_2_O). As the pH decrease continues, close to 12, the phase 4CaO-Al_2_O_3_−13H_2_O is dissolved. Subsequently, in an interval of 11.6 to 10 pH, the gypsum (CaSO_4_-2H_2_O) and gibbsite (Al(OH)_3_) precipitate at the expense of the dissolution of ettringite. Finally, when close to pH 8, the CSH gel dissolves, and amorphous silica (SiO_2_) is formed [54]. This sequence represents changes in concrete or mortar that are subjected to an environment conducive to carbonation. The findings of this investigation on the changes in chemical stability and on the surface condition of the sugarcane bagasse fibers as the pH varies are presented in Table 2. 

In a fresh mortar with a pH greater than 12, the matrix maintained the stability of the hydrated calcium silicates and therefore the chemical properties, while the fibers broke down into smaller units. A decrease in pH promoted reactions that generated new compounds in the matrix, while in the cane bagasse fibers, the decrease in pH only caused them to acquire a greater volume. Contrary effects were noted in a very alkaline pH, where the matrix remained in good condition, but the fiber was affected; by lowering the pH, the matrix reacted, and the fiber presented morphological but not chemical changes.

#### 3.2.3. Volume of Permeable Voids

Table 3 shows the volume of permeable voids for the samples. The decrease in voids is inverse to the increment of sugarcane bagasse fiber. This reduction of voids is due to the space occupied for the fibers, which is a disadvantage to the carbonation front advance. It is clear that a small difference between the percentage of permeable voids with an increment of the fiber content, negatively modified the capacity of reduction to carbonation in the mortars samples. This finding confirmed the sealing pores mechanism proposed in previous sections.

The performance tests indicated that the addition of sugarcane bagasse fiber improved the properties of the mortars. According to the results, the fiber deteriorated with the pH of the freshly cast mortar, but it is proposed that its decomposition generated smaller derivatives that increased its volume in the pore solution and sealed the porosities. As this was a process that was carried out throughout the mortar paste, it was assumed that the advance of the carbonation process from the surface was reduced.

## 4. Conclusions

Subjecting the bagasse fibers to alkaline pHs, such as the one that occurs during the mortar carbonation process, has a series of effects on the fibers according to the specific pH value to which it is exposed. When the pH is highly alkaline (pH 11 and 12), the fibers undergo delignification to form smaller chemical units, which is corroborated by the loss of the characteristic lignin peaks in the FTIR spectra of the samples subjected to these values of pH. In weak alkaline environments (pH 9 and 10), what happens is that the hydroxyl ions are added to the structure of the bagasse fiber components, causing the solubilization and separation of the hemicellulose from the cellulose.

Both phenomena can cause structural changes to the bagasse fibers, as was observed by the SEM analysis. In a neutral pH, the non-fibrous materials appear to be disordered and swollen because of the gain of hydroxyl ions. However, at pH 12 after 30 days of exposure, microfibrils of cellulose can be observed, because the lignin and hemicellulose layers that cover them are hydrolyzed and solubilized under these exposure conditions, which leads to the defibrillation of the fibrils, leaving the cellulose microfibrils more exposed to progressive degradation.

With the dispersion of fibers in the cementitious matrix, the mortars acquire a greater compressive strength. The effect of increasing the compressive strength when augmenting the fiber concentration is remarkable, up to 50% after adding 2% bagasse. In turn, that compressive strength increases over time. However, at 2% bagasse, the resistance after 90 days decreases, possibly because of the decomposition of fibers when exposed to an alkaline environment, but it still has a better compressive strength. The sugarcane bagasse fiber concentration reduces the penetration of CO_2_ and thus the carbonation of mortar from 35 mm of the control sample to 2 mm for the 2% bagasse fiber addition sample.

Both the better compressive strength and the reduction in the carbonation front for samples with cane bagasse added can be explained with the fibers behavior results of the FTIR and SEM analyses of fibers submitted to alkaline pH values of mortars, as it is observed that the fibers suffer a breakdown and an increment in their volume, causing a pore sealing effect in the mortar pastes. Because of this, the addition of cane bagasse fiber contributes to an enhancement in the durability properties of the manufactured mortars.

## Figures and Tables

**Table 1:** Components of the mortar samples.

Cement Components (%)						
	Clinker Portland + Calcium Sulfate	Granulated Blast Furnace Slag	Pozzolanic Materials	Sílica Fume	Limestone	Minority
CPC Maya [28]	50–94	6–35	6–35	1–10	6–35	0–5
Fine aggregate						
	Loose dry volumetric weight (kg/m^3^)	Dry rodded volumetric weight (kg/m^3^)	Density or specific gravity (gr/cm^3^)	% Absorption	Fineness modulus	% Humidity
Properties	1335	1514	2.56	5	2.5	3
	Mesh number	Diameter (mm)	Retained material (gr)	Retained material %	Accumulated Retained Material (%)	Passing material (%)
Granulometric analysis [29]	6	3.35	34.9	11.65	11.65	88.35
	8	2.36	49.7	16.59	28.24	71.76
	30	0.6	72	24.04	52.28	47.72%
	50	0.3	52.8	17.53	69.91	30.09%
	100	0.15	40.3	13.45	83.36	16.64%
	200	0.075	46.2	15.43	98.7	1.21%
	Tray		3.6	1.21	100	0

**Table 2:** Chemical and morphological changes in bagasse fibers as a result of pH.

pH	Effect on Sugarcane Bagasse Fibers
12–11	They lose their peak characteristic of cellulose at 1430 cm^−1^ as well as the characteristic peak of lignin, which corresponds to delignification.
10–7	More voluminous. Hydroxyl ions are added to the structure of the bagasse fiber components, causing molecule swelling, hydrolysis of the ester bonds, and breaking of the inter-molecular hydrogen bonds between hemicellulose and cellulose, causing the hemicelluloses to become solubilized in water and separating from the cellulose fibers.

**Table 3:** Percentage of volume of permeable voids as a function of sugarcane fiber content.

% Sugarcane fiber	0	0.5	1.0	2.0
% Volume of permeable voids	27.18	26.31	25.58	23.69

## Data Availability

Data is contained within the article.
